# Pulmonary artery sarcoma masquerading as pulmonary embolism: the pivotal role of sonographic differentiation — a case report

**DOI:** 10.3389/fonc.2026.1709231

**Published:** 2026-01-21

**Authors:** Yahong Wang, Peng Zhao, Wanyan Li, Lihong Wang

**Affiliations:** 1 School of Medical Imaging, Shandong Second Medical University, Weifang, Shandong, China; 2 Department of Cardiac Surgery I, Yantai Yuhuangding Hospital Affiliated to Qingdao University, Yantai, Shandong, China; 3 Department of Ultrasound, Yantai Yuhuangding Hospital Affiliated to Qingdao University, Yantai, Shandong, China

**Keywords:** computed tomography angiography (CTA), echocardiography, magnetic resonance imaging (MRI), positron emission tomography-computed tomography (PET-CT), pulmonary artery sarcoma (PAS), pulmonary embolism (PE)

## Abstract

Pulmonary artery sarcoma (PAS) is a rare and highly aggressive malignancy. It often presents with nonspecific clinical symptoms and is frequently misdiagnosed as pulmonary embolism (PE). Studies suggest that approximately 50% of PAS cases are initially misdiagnosed as PE. The discovery in this case report was an incidental finding. We describe the case of a patient admitted with chest tightness and chest pain. Due to a history of coronary stent implantation for 10 years, acute coronary syndrome was initially considered. Echocardiography was carried out primarily to assess cardiac condition. However, the examination revealed a hypoechoic mass in the main pulmonary artery with morphological features atypical for PE. Computed tomography angiography (CTA) was therefore recommended for further evaluation. Although the initial CTA interpretation suggested PE, the discordance between the echocardiographic and CT findings prompted the immediate initiation of anticoagulation therapy alongside a concurrent multidisciplinary consultation. The consensus following the discussion indicated a high probability of PAS. The patient subsequently underwent surgery at another hospital, and the postoperative pathology confirmed PAS. To our knowledge, current reports on PAS focus predominantly on findings from CTA, MRI, and PET-CT, while echocardiographic features are often only mentioned cursorily. However, echocardiography offers a unique capability for dynamic assessment of the tumor, which is not available with other imaging modalities. The aim of this report is to enhance the understanding of the echocardiographic manifestations of PAS, thereby facilitating earlier detection and potentially creating opportunities for surgical intervention.

## Introduction

Pulmonary artery sarcoma (PAS) is a rare, highly aggressive malignancy arising from pluripotent mesenchymal cells in the pulmonary artery intima (1). While PAS are histologically divided into intimal and luminal subtypes, the latter occurs very infrequently. In clinical practice, PAS almost exclusively denotes intimal sarcoma (2, 3). It often presents with nonspecific clinical symptoms and is frequently misdiagnosed as PE (4). Studies suggest that approximately 50% of PAS cases are initially misdiagnosed as PE (5). PAS progresses rapidly, causing luminal obstruction and early metastasis.At the time of diagnosis, approximately 50% of patients have pulmonary metastases, 16% to 19% have distant metastases, and about 60% experience recurrence (2). This study presents a case of a 67-year-old Asian male presenting with chest tightness and pain, in whom a routine echocardiogram incidentally revealed a main pulmonary artery mass. The lesion was subsequently resected and histologically confirmed as PAS. We emphasize the laboratory findings and imaging characteristics of PAS, with a particular focus on its sonographic features. This analysis aims to improve disease recognition and facilitate a timely and accurate diagnosis.

## Case description

**Figure 1:**
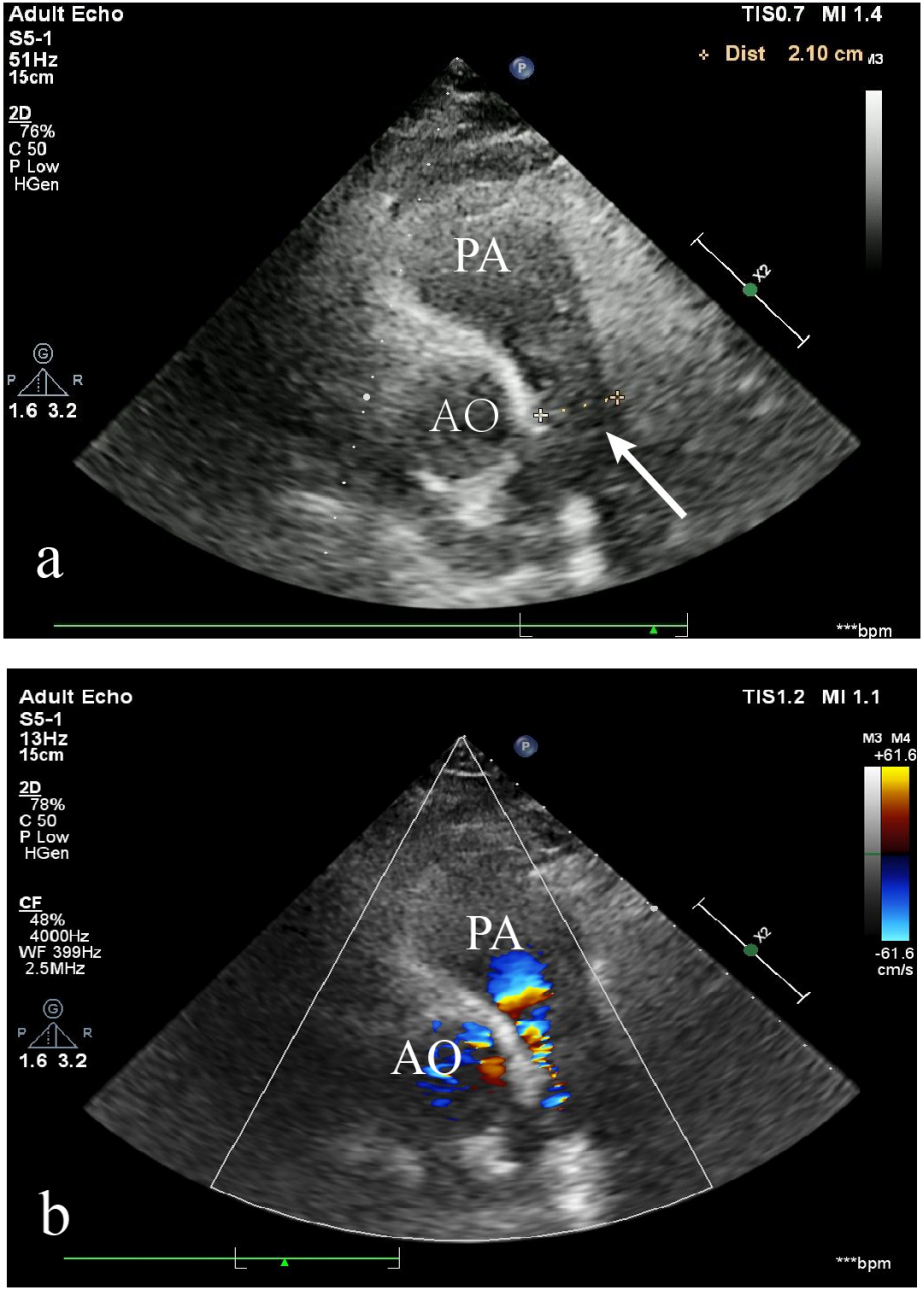
Transthoracic echocardiogram (parasternal short-axis view at the level of the great arteries). Abbreviations: AO, aorta; PA, pulmonary artery. **(a)** A hypoechoic mass (demarcated by white arrowheads) is visualized within the pulmonary artery, nearly occupying its entire lumen. **(b)** Color Doppler flow imaging demonstrates an eccentric, high-velocity, forward-flow jet across the obstruction within the narrowed residual lumen.

**Figure 2:**
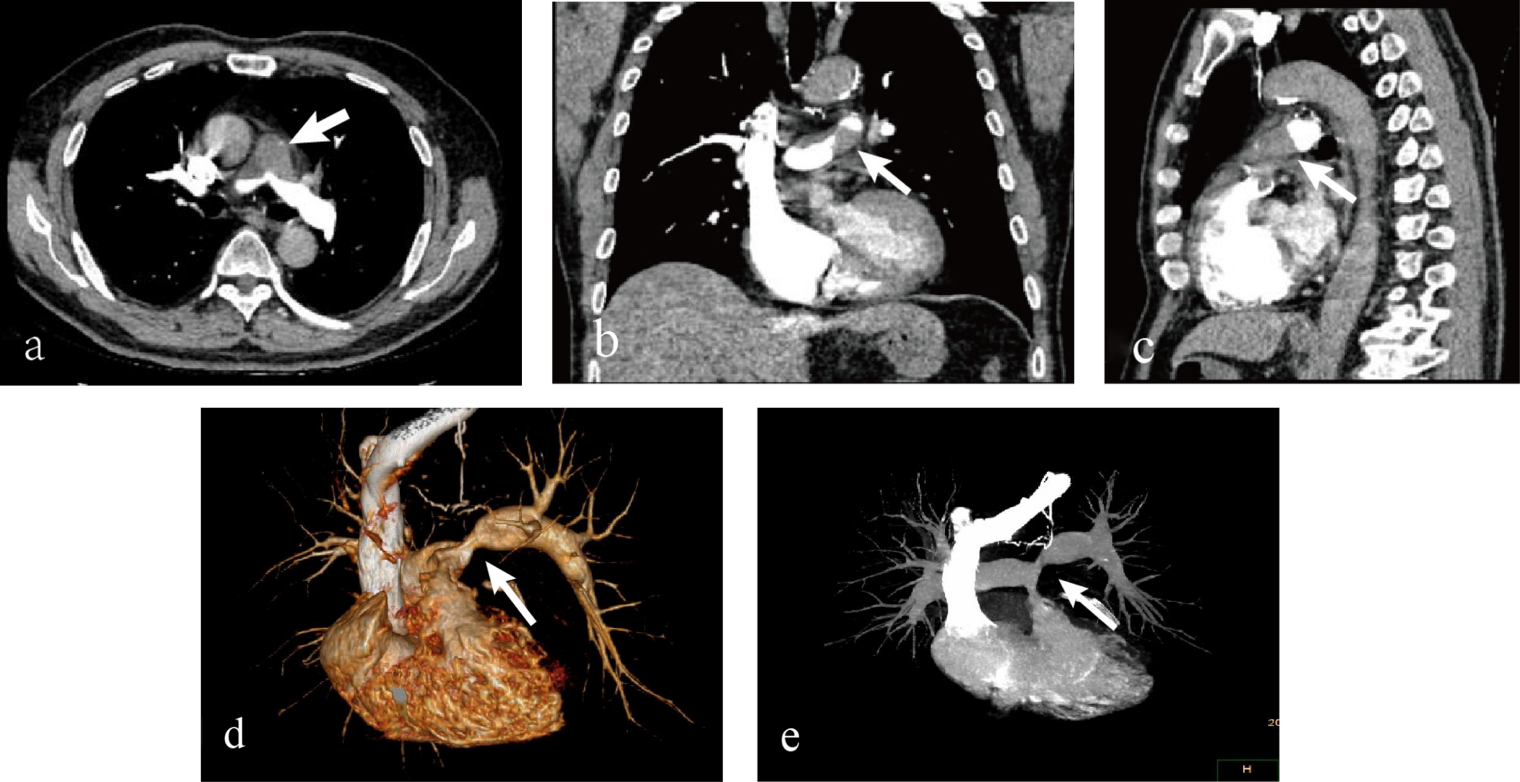
Computed tomography angiography (CTA) findings. **(a)** Axial contrast-enhanced CT image reveals a well-defined filling defect (arrow) occupying most of the main pulmonary artery lumen. **(b)** Coronal reformatted image better demonstrates the extent of the mass and its irregular, lobulated border (arrows). **(c)** Sagittal view further confirms the presence of the filling defect (arrow) within the pulmonary artery. The multi-planar reconstruction comprehensively delineates the lesion. **(d)** Three-dimensional volume-rendered image of the mass and pulmonary vasculature. **(e)** Maximum intensity projection (MIP) image highlighting the filling defect within the contrast-opacified pulmonary artery.

**Figure 3:**
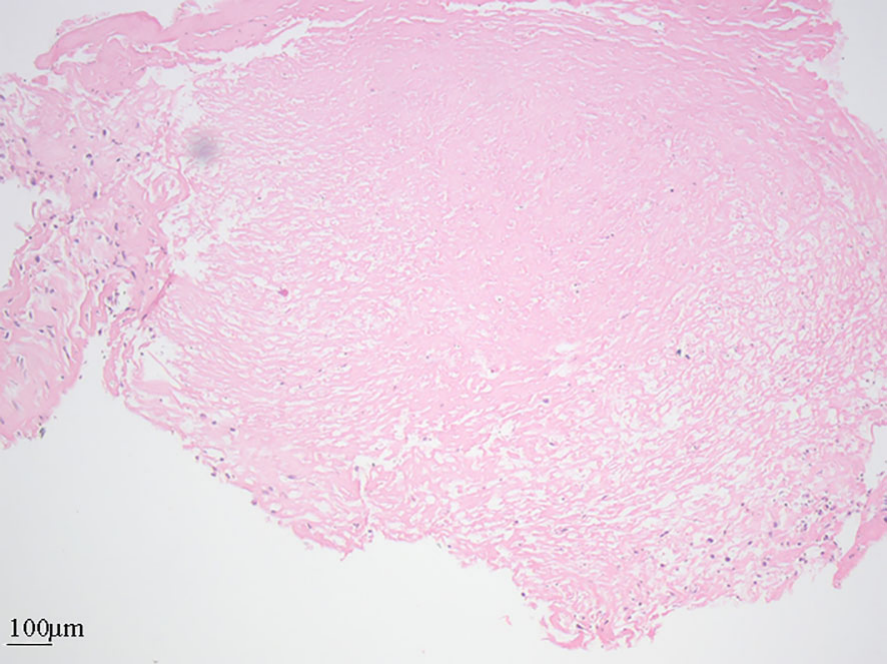
Histopathological examination of the biopsy specimen (hematoxylin and eosin staining). The tissue is largely composed of coagulative necrosis, bordered by focal areas of atypical cells.

A 67-year-old Asian male presented with a five-year history of episodic chest tightness and pain, which had aggravated over the past month. Specifically, the patient experienced a recurrence of symptoms one month ago without apparent cause. The chest pain was irregular, showed no consistent pattern in relation to activity, and usually subsided within a few minutes, whether it occurred at rest or after exertion. No cough or sense of impending doom was reported. Ten years ago, coronary angiography demonstrated severe stenosis, necessitating coronary stent implantation. He has remained on oral antiplatelet therapy since the procedure. He also had a 10-year history of hypertension, which was well-controlled with antihypertensive medications. The patient reported no significant family history. Physical examination revealed blood pressure was 118/78 mmHg (reference range: 90-139/60-89), heart rate was 86 bpm (reference range: 60–100 bpm), and a grade III/VI systolic murmur at the tricuspid valve area. The initial diagnostic impression based on the medical history was acute coronary syndrome. Subsequent diagnostic evaluation comprised electrocardiography, laboratory studies, and echocardiography. Electrocardiographic findings may remain normal even during episodes of chest pain. Laboratory investigations revealed an elevated B-type natriuretic peptide (BNP) level of 199.34 pg/mL (reference range: 0.00-100.00 pg/mL) and a mild elevation in creatine kinase-MB isoenzyme (5.5 ng/mL, reference range: 0.00–4.94 ng/mL). Myoglobin, troponin, D-dimer, prothrombin activity, and thrombin time were all within normal limits. Echocardiography demonstrated right atrial and ventricular enlargement, a 38×12 mm hypoechoic mass in the main pulmonary artery (**Figure 1a**), associated with stenosis, eccentric accelerated forward flow on color Doppler (**Figure 1b**), mild pulmonary regurgitation, and severe tricuspid regurgitation. These findings were atypical for PE; therefore, further evaluation with CTA was advised. The presence of a filling defect in the main pulmonary artery on CTA was interpreted as supporting a diagnosis of PE (**Figure 2**). The patient was prescribed an anticoagulant, a diuretic, an antihypertensive agent, and medications for pulmonary arterial hypertension. Following the treatment, the patient exhibited no significant symptomatic improvement. Hence, the cardiology specialists conducted a comprehensive literature review and convened a multidisciplinary consultation involving specialists from radiology, ultrasonography, cardiology, cardiac surgery, radiation oncology, rheumatology, and oncology. Following comprehensive evaluation, the clinical presentation was deemed inconsistent with PE, raising suspicion for a pulmonary artery tumor. The diagnostic rationale was outlined as follows: (1)The patient exhibited an insidious onset with progressive worsening over one month, contrasting with the acute chest tightness and pain characteristic of pulmonary embolism. (2)D-dimer levels remained within normal limits, deviating from the elevated values typically observed in thromboembolic events. (3)Imaging studies revealed a predominant lesion in the main pulmonary artery trunk, with complete sparing of distal lobar and segmental branches. For a definitive diagnosis, surgical intervention was recommended for the patient. The patient opted to seek treatment at a another tertiary hospital. Subsequent follow-up via telephone interview revealed that the patient had undergone “pulmonary artery tumor resection, right ventricular outflow tract reconstruction, pulmonary valve replacement, and pulmonary arterioplasty” at another hospital.” Surgical records indicate the intraoperative findings of tumor tissue filling the pulmonary artery and right ventricular outflow tract, with indistinct demarcation between the neoplastic tissue and cardiac walls. The procedure involved resection of the right ventricular outflow tract musculature, complete excision of the main pulmonary artery with partial resection of the right and left pulmonary arteries, followed by interposition of an artificial vascular conduit to connect the bilateral pulmonary arteries. A homograft aortic valve was utilized to connect the right ventricular outflow tract prosthesis, with additional pericardial patch augmentation of the right ventricular outflow tract. Histopathological examination revealed focal destruction of endomyocardial tissue with preserved smoothness of the pulmonary arterial walls. Postoperative pathology confirmed undifferentiated pleomorphic sarcoma invading the subendocardial myocardium, with involvement at the distal margin. The patient declined adjuvant chemotherapy or radiotherapy. One year postoperatively, he presented with dyspnea. Repeat CTA revealed filling defects in the right ventricular outflow tract and left pulmonary artery, suggestive of tumor recurrence. Pulmonary angiography and transcatheter biopsy confirmed recurrence, with pathologic findings of coagulative necrosis and scattered atypical cells (**Figure 3**). The patient again declined treatments such as chemotherapy or radiotherapy and received symptomatic treatment only. Follow-up via telephone interview after discharge indicated that the patient died 40 days after hospitalization.

## Discussion

Pulmonary artery sarcoma (PAS) is a rare, highly aggressive malignancy arising from pluripotent mesenchymal cells in the pulmonary artery intima (1). While pulmonary artery sarcomas are histologically divided into intimal and luminal subtypes, the latter occurs very infrequently; thus, in clinical practice, PAS almost exclusively denotes intimal sarcoma (2, 3). Intimal sarcomas are neoplastic lesions of the intimal layer in large vessels, primarily the pulmonary artery and aorta, characterized by endoluminal growth that leads to vessel obstruction or distal embolization (1). Since Mandelstamm’s first autopsy report in 1923, only approximately 400 cases have been reported in the medical literature. Epidemiological data indicate an incidence of 0.001%–0.03%, with a broad age range (13–86 years) but a predominance in middle-aged individuals. A slight female predominance exists (female-to-male ratio approximately 1.3:1). PAS progresses rapidly, causing luminal obstruction and early metastasis (1, 2, 4). At diagnosis, about 50% of patients exhibit pulmonary metastases, and about 16% have distant metastases (2). Untreated patients have a median survival of 1.5 months, while those receiving aggressive treatment survive up to 17 months (6). Clinical manifestations are nonspecific, including dyspnea, chest pain, dry cough, hemoptysis, palpitations, arrhythmias, weight loss, anemia, and fever (5–7). Diagnosing PAS is challenging, as it is frequently misdiagnosed as PE, leading to delayed management. Studies indicate that approximately 50% of PAS cases are initially misdiagnosed as PE, and these patients often receive anticoagulant therapy. The diagnosis of PAS is typically confirmed only after anticoagulation proves ineffective, prompting further investigation (7). Early diagnosis of PAS is therefore crucial for prolonging survival and preserving quality of life. The diagnostic evaluation of PAS typically incorporates multiple imaging modalities, including echocardiography, CTA, and MRI, as well as supplementary techniques such as PET-CT (2, 7). Among these, echocardiography serves as the first-line imaging modality for evaluating PAS in patients presenting with chest pain or dyspnea. This technique provides distinct advantages in assessing tumor dynamics and is widely employed for initial PAS screening (3). To enable more precise clinical differentiation, we conducted a comparative analysis of laboratory parameters and echocardiographic findings across three conditions: PAS, acute PE, and chronic PE. The principal findings are summarized below: (1) D-dimer: The interpretation of D-dimer levels in PAS requires nuance. While the marked elevation characteristic of acute PE is typically absent, approximately 41.0% of PAS patients demonstrate a mild elevation in D-dimer levels (8). Furthermore, the clinical scenario can be complicated by the occasional coexistence of PAS and PE (8). Therefore, although a normal or mildly elevated D-dimer does not exclude PAS, a level below 2.81 μg/mL has been suggested to increase its diagnostic likelihood relative to PE in the appropriate clinical context (1). (2) NT-proBNP: Levels are lower in PAS compared to acute/chronic PE, reflecting milder right ventricular dysfunction (9). (3) Echocardiography: a. Location: PAS predominantly involves the main pulmonary artery and major branches (85% main, 71% right, 65% left), with frequent involvement of the pulmonary valve (32%) and right ventricular outflow tract (10%)^(4)^. Central PE often affects bilateral arteries, whereas chronic PE more frequently involves unilateral arteries. b. Echogenicity: PAS appears as hypo-/isoechoic, heterogeneous masses with irregular margins (10), while PE is hypoechoic and chronic PE often becomes hyperechoic. c. Morphology: PAS typically demonstrates pulmonary artery dilatation containing an irregular, lobulated mass with extraluminal invasion, features that strongly support the diagnosis. In contrast, pulmonary PE characteristically presents as intraluminal filling defects with smooth margins (10). This patient presented with chronic-onset symptoms (chest pain and tightness), normal D-dimer, and mildly elevated NT-proBNP. Ultrasound revealed a ‘hypoechoic, irregular, nodular mass’ nearly occluding the main pulmonary artery, with eccentric high-velocity flow adjacent to the vessel wall. Distal branches (lobar/segmental) were unaffected. With no history of deep vein thrombosis and the presence of long-term antiplatelet therapy (following coronary stent implantation) — which further reduced the thrombotic risk — the findings collectively strongly supported a diagnosis of pulmonary artery tumor (specifically sarcoma) over thromboembolism.

Beyond ultrasonography, the integration of advanced imaging techniques—including CTA, MRI, and PET-CT—is critical for diagnosing PAS (2).

Key morphological features on CTA and MRI include: (1) invasion of the pulmonary valve or right ventricular outflow tract; (2) the vascular wall erosion sign (WES), and (3) a lobulated proximal mass or a distally located “cluster of grapes” appearance, often accompanied by proximal lobular morphology. On MRI, PAS typically appears hyperintense on both fat-suppressed T2-weighted and diffusion-weighted imaging (DWI) sequences, and demonstrates heterogeneous enhancement after contrast administration.

Functional imaging with PET-CT plays a pivotal role in differentiating PAS from benign PE. PAS exhibits significantly greater 18F-fluorodeoxyglucose (FDG) uptake than benign PE. Lee et al. reported that applying a maximum standardized uptake value (SUVmax) threshold of 3.5 allowed perfect differentiation between malignancy and PE (100% sensitivity, specificity, and accuracy) (9, 11). Moreover, PET-CT has also demonstrated diagnostic utility in cases where conventional CT findings are indeterminate, enabling the differentiation of PAS from other conditions that may mimic its radiological presentation (8).

The diagnosis of PAS has traditionally relied on surgical procedures; however, minimally invasive techniques such as endobronchial ultrasound-guided transbronchial cryobiopsy (11) and pulmonary angiography-guided cryobiopsy are now available. The latter modality was used to diagnose postoperative recurrence in this patient. Surgical resection remains the cornerstone of PAS management, with early intervention being critical for improving prognosis. There are no standardized protocols for adjuvant therapies (chemotherapy or radiotherapy) due to limited evidence.

Echocardiography is commonly used to screen for pulmonary artery space-occupying lesions in patients with chest pain or dyspnea. It is considered the first-line imaging modality for PAS evaluation (3). It should be noted that a recent pooled analysis highlights the diagnostic challenge in early-stage PAS: only a limited number of cases can be confidently diagnosed during the initial presentation due to variability in imaging features and differences in echocardiography operator expertise (8). Clinicians should suspect PAS when lesions primarily involve the large pulmonary arteries while sparing distal branches; when there is involvement of the right ventricular outflow tract or pulmonary valve; and when no thrombi are detected in the lower extremities or inferior vena cava. Additionally, a normal D-dimer and lack of improvement after anticoagulation further support the suspicion.

## Data Availability

The original contributions presented in the study are included in the article/Supplementary Material. Further inquiries can be directed to the corresponding author.
