# Supplementary figures and images for: Pulmonary artery sarcoma masquerading as pulmonary embolism: the pivotal role of sonographic differentiation — a case report
Source: Front Oncol. 2026 Jan 21;16:1709231. doi: 10.3389/fonc.2026.1709231 (PMC12867922; doi:10.3389/fonc.2026.1709231)

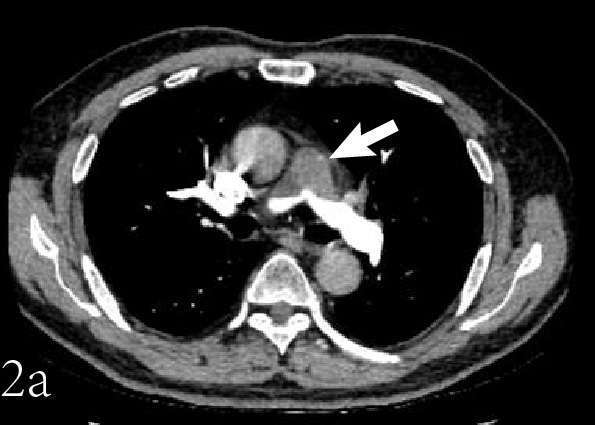

Supplement: Supplementary file 1 [file Image1.jpeg]

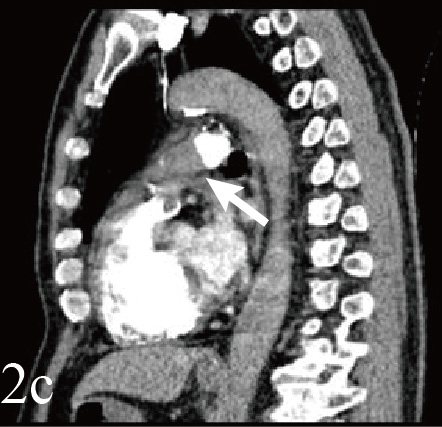

Supplement: Supplementary file 2 [file Image2.jpeg]

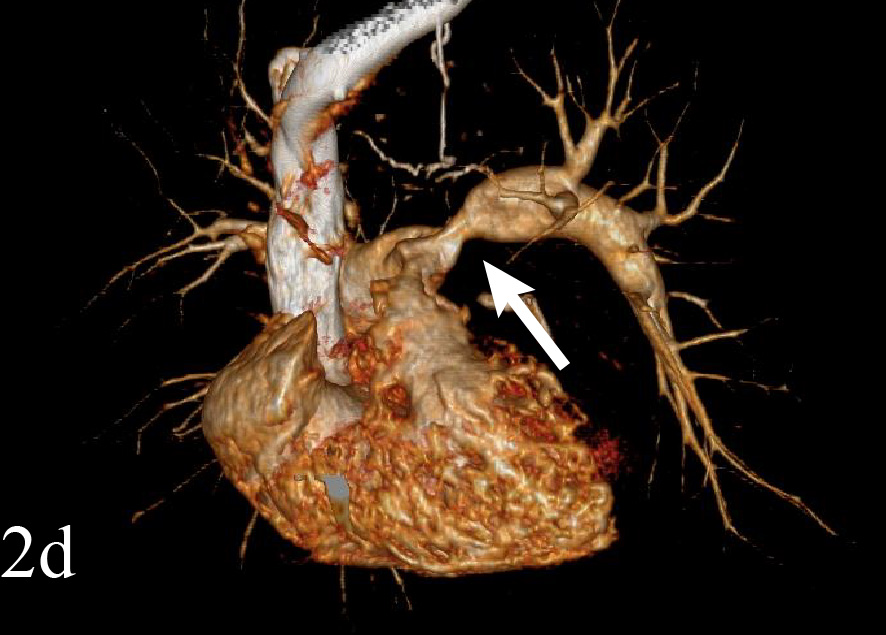

Supplement: Supplementary file 3 [file Image3.jpeg]

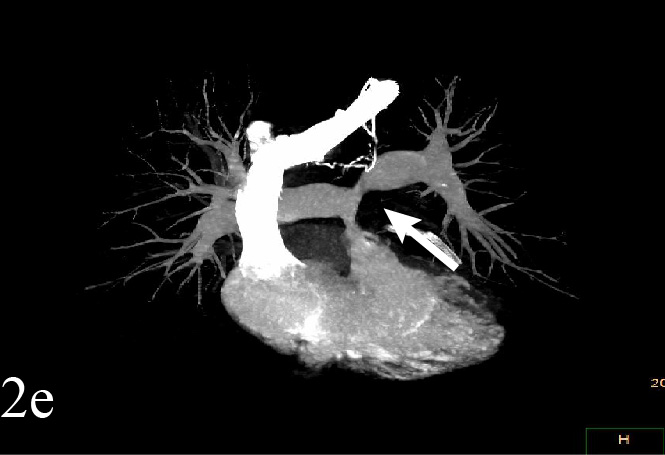

Supplement: Supplementary file 4 [file Image4.jpeg]
